# Analysis of serum levels of titanium and aluminium ions in patients with early onset scoliosis operated upon using the magnetic growing rod—a single centre study of 14 patients

**DOI:** 10.1007/s43390-021-00335-1

**Published:** 2021-07-23

**Authors:** Mandar Deepak Borde, Sarang Sapare, Emile Schutgens, Chadi Ali, Hilali Noordeen

**Affiliations:** 1 Department of Orthopaedics and Spine Surgery, Bharati Vidyapeeth (Deemed to be University) Medical College, Pune-Satara Road, Dhankawdi, Pune, Maharashtra 411043 India; 2 Department of Spine Surgery, Royal National Orthopaedic Hospital, Stanmore, Middlesex UK

**Keywords:** Early onset scoliosis, MCGR, Serum levels, Titanium ions, Aluminium ions

## Abstract

**Study design:**

A cross-sectional retrospective Level 3 study.

**Objective:**

To study the serum levels of Titanium and Aluminium ions in patients operated using the magnetically controlled growing rod (MCGR) system.

**Summary of background data:**

14 consecutive patients of early onset scoliosis with varying etiology managed with MCGR system with a minimum follow-up of 24 months were selected for the study. The group consisted of two boys (14.3%) and 12 girls (85.7%). The average age of the patients at the time of surgery was 10.4 years (5–15 years). The average period of follow-up was 43.7 months (28–79 months). After informed consent of the subjects and their caretakers, serum levels of titanium and aluminium were measured. These levels were then assessed with regards to the number of screws used, number of distractions and complications.

**Methods:**

The concentration of titanium and aluminium ions in the serum was measured using high resolution inductively coupled plasma mass spectrometry.

**Results:**

For the sake of ease of assessment, patients were divided into three etiology-based groups—idiopathic (*n* = 6), neuromuscular (*n* = 2) and syndromic (*n* = 6). The mean serum titanium level was 15.9 μg/L (5.1–28.2 μg/L) while that of aluminium was 0.1 μmol/L (0.1–0.2 μmol/L). Of the 14 patients, 2 (14.2%) patients had mechanical failure (actuator pin dysfunction), 3 (21.4%) had rod breakage requiring revision surgery and one patient (7.1%) had surgical site infection managed with appropriate antibiotics. Patients undergoing revision for rod breakage did not show any metallosis of the tissues during surgery.

**Conclusion:**

Analysis of patients with scoliosis operated using the magnetic growing rod system concludes that it is accompanied by presence of titanium in the blood but whether clinically significant or not needs to be ascertained by comparison of preoperative and postoperative blood concentrations of the titanium ions in individual subjects. The aluminium ion concentration remains within normal limits. Though implant malfunction may raise the titanium levels in the blood, its clinical significance needs to be determined. The aluminium levels are not affected irrespective to the presence or absence of complications. The long-term effects of raised titanium levels in the blood also warrant further prospective studies designed for precise and deeper analyses.

## Introduction

The treatment of early onset spinal deformities has consistently posed a challenge to the spinal surgeons for a long time. The advent of fusionless instrumentation that allows spine growth in addition to arresting the progression of deformity in cases with high growth potential has helped avoid early fusion surgery in paediatric patients. Growing rods (GR) act as temporary internal braces that allow the growth of the spine, lungs and the thoracic cage while controlling the deformity. These growing rods can be mechanically or magnetically extended [1, 2]. These growing rods require intermittent extension (atleast twice or thrice yearly). The magnetically extendable growing rods allow a non-invasive method of extension as compared to the mechanically extendable ones.

As these rods slide in the guiding fixtures, they have a potential to generate excessive metallic debris (metallosis). Metal ion release has been addressed in adolescent idiopathic scoliosis (AIS) literature. Localized metal debris has been well documented in the paraspinal soft tissues surrounding instruments [3]. Elevated levels of titanium (Ti) ion in the blood and its systemic distribution via blood and lymphatic vessels and distant organ settlement of particulate debris have also been demonstrated [4–6]. A case report identifying wear debris-induced osteolysis around a pedicle screw after posterior spine fusion in a pediatric patient has also been reported [7].

When compared to instrumented spinal fusion for AIS, GR applications comprise fewer implants but they are more frequently manipulated. Therefore, although the dose and mode may vary, logically thinking, children with early onset deformities treated with growing rods should also be subjected to metallic exposure. Yet, very less research has focused on metal-ion release and distribution in growth friendly instrumentation for early-onset scoliosis (EOS). Also, the clinical effects of the raised metal ion levels in children are yet to be determined. The aim of this study was to measure the content of titanium and aluminium ions in the blood of patients having high growth potential and treated with magnetically controlled growing rod (MCGR) system.

## Material and methods

### Study design and participants

This was a single centre, cross-sectional retrospective study. Fourteen consecutive patients (two boys and 12 girls) with early-onset scoliosis attending the Spine Deformity Clinic with an average age of 10.4 years (5–15 years) at the time of surgery and treated with magnetically controlled growing rod (MCGR) system during the period from 2010 to 2017 with a minimum postoperative follow-up of 24 months were recruited in the study group. The average time between the index surgery and the time the blood was drawn was 43.7 months (28–79). The subjects comprised of six patients with idiopathic scoliosis, two patients with neuromuscular scoliosis and six patients with syndromic scoliosis (Table 1). Of the 14 patients, patients with Cerebral palsy and those with Prader Wili syndrome, Andersen Tawil syndrome and Rett syndrome were unable to ambulate independently due to their primary syndromic condition. The remaining nine patients were independent ambulators. Twelve patients were implanted with the first generation MCGR while two received the newer generation rod. All patients underwent a dual rod construct. A total number of 111 screws were used at the anchor points with 7.9 screws per construct (range 6–11) (Table 2).

**Table 1:** Table depicts the demographic and etiological details with the serum concentrations of the titanium and aluminium ions of all the subjects

Patient number	Gender	Age at Surgery (years)	Duration of blood test since surgery (months)	Pre op coronal Cobb angle (degrees)	Pre op sagittal Cobb angle (degrees)	Length achieved at the end of all lengthnings (mm)	Etiology	Titanium (μg/L)	Aluminium (μmol/L)
1	M	5	38	78	77	17.8	Sotos syndrome	14.9	0.2
2	F	11	44	96	84	15.7	Rett syndrome	25.6	0.1
3	F	13	39	55	35	10.5	Idiopathic	5.0	0.1
4	F	11	29	93	25	10.5	Idiopathic	10.3	0.1
5	F	13	28	98	45	22.7	Pterygium syndrome	28.2	0.1
6	F	15	31	86	70	5	Cerebral Palsy	19.7	0.2
7	M	7	79	95	38	48.1	Prader Willi syndrome	14.5	0.2
8	F	12	40	70	24	90	Andersen Tawil syndrome	9.9	0.2
9	F	12	40	66	28	48.2	Idiopathic	13.1	0.2
10	F	7	60	87	47	49.5	Idiopathic	20.6	0.1
11	F	10	48	70	44	18.3	Cerebral Palsy	14.5	0.2
12	F	9	48	72	30	55.6	Idiopathic	12.7	0.2
13	F	12	40	65	42	16.8	Neurofibromatosis 1	18.7	0.1
14	F	9	48	55	16	22.5	Idiopathic	15.3	0.1

**Table 2:** Table depicting the number of screws per construct with the number of distractions and the corresponding serum levels of titanium and aluminium

Patient number	Number of screws per construct	Number of distractions	Complications	Titanium levels (μg/L)	Aluminium levels (μmol/L)
1	8	8	Rod breakage	14.9	0.2
2	10	4	Actuator pin dysfunction	25.6	0.1
3	8	2	None	5.0	0.1
4	6	7	None	10.3	0.1
5	10	6	Surgical site infection	28.2	0.1
6	11	4	Rod breakage	19.7	0.2
7	8	5	Actuator pin dysfunction	14.5	0.2
8	8	4	None	9.9	0.2
9	6	6	None	13.2	0.2
10	6	9	Rod breakage	20.6	0.1
11	8	9	None	14.5	0.2
12	8	6	None	12.7	0.2
13	6	7	None	18.7	0.1
14	8	9	None	15.3	0.1

The patients underwent a total of 75 distractions with an average of 5.3 distractions per patient (range 2–9) (Table 2) at 3-month intervals in the outpatient department during their regular follow-up.

The study was approved by the Institutional Ethical Committee.

### Collection and analysis of specimens

The MCGR comprises of Ti-6Al-4 V ASTM F136 titanium alloy.

After appropriate consent of the subjects and their care takers, venous blood samples were collected after the lengthening procedure during the regular follow-up. The serum titanium and aluminium levels were measured using high-resolution inductively coupled plasma mass spectrometry.

5 mL of blood were collected into Vacuette® Trace Elements tubes (Greiner Bio-One International) coated with sodium heparin as anticoagulant. The samples were refrigerated at 4 °C prior to analysis. Samples were analysed for titanium and aluminium content on a Thermo Element 2 h ICP-MS instrument (Thermo Fisher Scientific GmBH, Bremen, Germany). 150 μL of each sample were dispensed into polystyrene assay tubes with 150 μL of water and 4.5 mL of assay diluent (0.5% (v/v) tetramethylammonium hydroxide (Electronics grade, Alpha Aesar, US), 0.005% (v/v) Triton X-100 (Romil, UK)) and 2.5 μg/L gallium (Alpha Aesar, US). Calibration standards were prepared by dilution from a custom stock solution (Qmx Laboratories Limited, Thaxted, UK) with a titanium and aluminium concentration traceable to NIST SRM 3162a Lot 130,925. The standard concentrations were: 0.00, 1.00, 4.00, 9.99, 39.96, 99.90 and 249.75 μg L^−1^. Serum matrix matched calibrations were prepared by dispensing 150 μL of each standard into polystyrene assay tubes with 4.5 mL of assay diluent and 150 μL and either Defibrinated Horse Blood (TSC Biosciences, Buckingham, UK) or Foetal Bovine Serum (Sigma-Aldrich, UK).

### Statistical methods

The mean value of the titanium and aluminium ions concentrations were calculated. There is no universally accepted minimum and maximum range of serum levels of titanium ions in the paediatric population. The range of serum aluminium level quoted by the institutional laboratory was 0–0.4 μmol/L. The patients were divided into three groups based on their etiology of idiopathic (*n* = 6), neuromuscular (*n* = 2) and syndromic (*n* = 6) (Table 3) for the ease of assessment. In addition, patients with and without any complications were divided into two groups.

**Table 3:** Etiology wise distribution of the serum titanium and aluminium levels

Patient number	Titanium level (μg/L)	Aluminium level (μmol/L)
Idiopathic group		
3	5.0	0.1
4	10.3	0.1
9	13.1	0.2
10	20.6	0.1
12	12.7	0.2
14	15.3	0.1
Neuromuscular group		
6	19.7	0.2
11	14.5	0.2
Syndromic group		
1	14.9	0.2
2	25.6	0.1
5	28.2	0.1
7	14.5	0.2
8	9.9	0.2
13	18.7	0.1

## Results

The mean serum titanium levels were 15.9 μg/L (5.1–28.3) while that for aluminium were 0.1 μmol/L (0.1–0.2). The mean titanium levels of the idiopathic, neuromuscular and syndromic groups were 12.9 μg/L (5.1–20.6); 17.1 μg/L (14.5–19.7) and 18.6 μg/L (9.9–28.2), respectively, while those for aluminium were 0.1 μmol/L (0.1–0.2); 0.2 μmol/L (0.2–0.2) and 0.1 μmol/L (0.1–0.2), respectively (Table 3). In the idiopathic group (*n* = 6), one patient had to undergo a revision surgery for a rod breakage (titanium level 20.6 μg/L; aluminium level 0.11 μmol/L). The neuromuscular group (*n* = 2) comprised of cerebral palsy patients. One of these patients underwent a revision surgery for a rod breakage (titanium level 19.7 μg/L; aluminium level 0.2 μmol/L). The syndromic group (*n* = 6) comprised one patient each of Sotos syndrome (SS), Rett syndrome (RS), Prader Willi syndrome (PWS), Neurofibromatosis 1 (NF1), Andersen Tawil syndrome (ATS) and Pterygium syndrome (PS). Of these, patients with RS (titanium level 25.6 μg/L; aluminium level 0.1 μmol/L) and PWS (titanium level 14.5 μg/L; aluminium level 0.2umol/L) suffered mechanical failure in the form of actuator pin dysfunction; patient with PS (titanium level 28.2 μg/L; aluminium level 0.1 μmol/L) had a surgical site infection managed with appropriate antibiotics and the SS patient (titanium level 14.9 μg/L; aluminium level 0.2 μmol/L) suffered rod breakage warranting revision surgery. The mean serum titanium level of patients experiencing complications was 20.6 μg/L (14.5–28.2) which was higher than the mean level of those without any complications 12.5 μg/L (5.0–18.7). The mean serum levels of patients experiencing complications and those without were almost the same (15.4 and 15.3 μmol/L, respectively).

None of the patients who underwent revision surgery showed any signs of metallosis of the tissues surrounding the rods.

## Discussion

AIS and arthroplasty literature suggest a systemic spread of the metals due to wear and tear of the implants [8–14]. One could argue that the metal release due to implantation would be lower in fusionless surgery because it comprises fewer implants and less intraoperative manipulation. Intersegmental motion might cause increased release due to wear as the implants sustain major load due to their fusionless nature. Moreover, additional metal ion release can be expected whilst repeated lengthenings [15, 16].

Titanium is also a micronutrient present in the body and majority of it is stored intracellularly [19]. Normal values for whole blood titanium levels in adult controls were 50–150 μg/L according to Synlab/Leinfelden, Germany [19]. Cundy et al., Richardson et al. and Kasai et al. have proven that the serum concentrations of Titanium ion in patients with Titanium-based spinal implants have been found to be on the higher side as compared to those of controls [4–6]. Swiatkowska et al. [20] conclude in their study that due to inter-laboratory analytical differences, reliable conclusions regarding "normal" and "abnormal" titanium levels in patients with orthopaedic implants are difficult to draw. Diagnosis of symptomatic patients should be based on radiographic evidence combined with blood/serum metal levels. There are no studies that mention universally accepted normal values for serum titanium levels in paediatric patients. Aluminium, on the other hand, is an essential micronutrient in the human body and is ubiquitous in its distribution [4]. There is constant exposure to this element through ingestion of water and food and exposure to dust particles [4]. None of the patients in the present study showed any clinical symptoms that could be attributed to the raised titanium levels in the serum. None of the patients showed raised levels of aluminium ions in their serum samples consistent with Cundy et al. [4]. All the patients were residents of the same geographical region and hence were exposed to the same amount of non-implant-related metallic exposure. All the samples were tested in the same laboratory and all the storage and processing modalities were identical for all the samples. None of the patients that underwent revision surgeries for implant related complications such as broken rods or actuator dysfunction showed any signs of metallic staining of the surrounding tissues (metallosis).

In cohorts of pediatric patients with a mean postoperative time of 6 years after stainless steel spinal instrumentation, Kim et al. [9] and Cundy et al. [17] reported time-dependent inverse relationships between serum metal levels (chromium) and time since spinal surgery. This “wearing-in” phenomenon has been attributed to the time taken for spinal fusion to occur (up to 24 months) and the associated decrease in micromotion between interfaces, which would limit wear and corrosion. The difference in our study is that the implant was not made of stainless steel but titanium-aluminium alloy. Whether titanium and aluminium levels also follow the above mentioned principle needs to be investigated. Patients with complications have shown elevated serum titanium levels compared to those without but whether these are clinically significant need to be determined. The levels of aluminium did not show any difference irrespective of presence or absence of complications. There is no linear correlation observed between the number of distractions or the number of anchor screws and the serum titanium and aluminium levels as can be seen in Table 2. The assumption that more active children may generate more wear debris than non-ambulatory children is not consistent as seen in the study that non-ambulatory Rett syndrome and Cerebral palsy patients have shown elevated titanium levels as consistent with the more active idiopathic scoliosis subjects. (Table 1).

There are certain limitations of this study. Ideally speaking, the MCGR system is indicated for skeletally immature patients less than 10 years of age with progressive spinal deformity. All the patients included in the study were diagnosed before 10 years of age. The patients operated after 10 years of age were still skeletally immature (Risser grade 0–1) and the girls were premenarchal with significant growth potential at the time of surgery. No controls were used in this study to compare the blood levels of the titanium and aluminium ions. Also, epidemiological data stating the normal blood concentration of the titanium ions in this population are not available. The MCGR system is a titanium, aluminium, vanadium and niobium alloy. Laboratory testing of titanium and aluminium is widely available as compared to that for vanadium and niobium as vanadium and niobium are not as widely present in nature. Should the titanium and aluminium levels had pathologically increased, the authors would have utilized the resources for testing vanadium and niobium levels. This is a cross-sectional study with a small sample size that analysed a time-dependant event. Ideally, the preoperative, intraoperative and immediate postoperative serum samples of the patients in addition to the serial serum concentrations of the titanium and aluminium ions of each subject should have been studied. Our study fails to compare the postoperative serum titanium and aluminium concentrations with those preoperatively; and therefore falls short as compared to the findings of Yilgor et al. that there is a time-dependant increasing trend in the blood concentrations of metal ions in patients of early onset scoliosis treated with growth friendly instrumentation [18].

Further large-sized, prospective and long-term studies need to be designed to analyse the preoperative levels of titanium and aluminium ions and then compare these with the postoperative levels over a long term in a periodic manner to get an in depth analysis of the subject.

## Conclusion

To conclude, implantation with magnetically controlled growing rod (MCGR) is accompanied by presence of titanium ions in the blood. The clinical significance of the same is yet to be determined. The serum aluminium levels tend to remain within normal limits. To our knowledge, the patients in our study have not shown any untoward clinical effects that could be attributed to the titanium levels in the serum. In addition, patients undergoing revision surgery for implant related complications such as broken rods or dysfunctional and broken actuators have not shown any signs of metallosis. Although, patients with broken rods and dysfunctional actuators have shown elevated serum titanium ion levels than those without these complications, determination of its clinical significance needs large-sized and long-term studies. There is no linear correlation between the number of anchor screws or the number of distractions and the serum titanium or aluminium concentrations.
